# Observations on the Prognostic in Acute Diseases

**Published:** 1782

**Authors:** 


IV. *Obſervations on the Prognoſtic in acute diſeaſes.* *By* Charles Le Roy, M. D. F. R. S. *Regius Profeſſor of Phyſic in the Univerſity of Montpellier, and Member of the Royal Society of Phyſicians at Paris. Tranſlated from the French, with Notes.* 8vo. Wilkie, *London*, 1782. 342 pages. 5 s.

THE art of prognoſticating in phyſic has always been conſidered as a very eſſential proof of a phyſician's knowledge, and his abilities in his profeſſion are very often meaſured by his ſkill in this department; nor is this altogether without reaſon, becauſe no man can foreſee what is likely to happen in an acute diſeaſe, who is not thoroughly acquainted with its nature, and at the ſame time able to diſtinguiſh, with accuracy, all the ſigns it affords, and then, after tracing each of theſe to its moſt probable cauſe, to weigh theſe cauſes with preciſion, and compare them with each other, and with the natural powers of the patient. The prognoſtic has therefore an immediate affinity with the diagnoſtic, and this knowledge evidently requires an extenſive acquaintance with books and nature. A phyſician, who excels in this art, muſt have ſtudied diſeaſes, both in his cloſet and at the bed-ſide of the ſick. He muſt likewiſe poſ-

ſeſs a clear, ſteady, and penetrating judgment, which ſuffers no ſymptom, nor even the moſt minute one to eſcape him, and which knows how to give to each ſymptom its juſt value. Theſe are qualities which every practitioner does not poſſeſs, and even they who do, will probably not be diſpleaſed to have an uſeful compendium like the preſent, which may facilitate their ſtudies in this way.

In the preface to this work, which is written by the tranſlator, and from which the preceding obſervations are extracted, it is remarked that the ancients, who ſtudied nature in herſelf, were extremely attentive to the ſigns of diſeaſes, and of courſe were well ſkilled in the art of prognoſticating. Semeiology, or the doctrine of ſigns, was confeſſedly the beſt and moſt uſeful part of the medical knowledge of the ancients. Phyſicians have hitherto ſtudied the prognoſtics in the writings of Hippocrates, but many of them are allowed to be ill founded, and others are altogether unintelligible, and have even been the ſubject of controverſy.

Proſper Alpini's ſeven books *de preſagienda vita & morte* have long been in great repute with phyſicians. Whoever reads them, ſees at once all the doctrine of the ancients on this ſubject; ject; but he copied from them indiſcriminately, without diſtinguiſhing between truth and error.

The author of the work before us, we are told, has carefully compared the prognoſtics of Hippocrates with his own obſervations; adopting ſuch as were conformable to truth, and correcting or rejecting ſuch as were defective or erroneous.

The work is divided into four ſections. In the firſt the author treats of the ſigns which indicate the ſtate of the circulating powers and viſcera. In the ſecond he deſcribes the evacuations, depoſitions, and eruptions that occur in acute diſeaſes. The third he allots to ſuch ſymptoms as could not with propriety be arranged under either of the preceding heads; and in the fourth he treats of inflammation and abſceſs of the breaſt, and ſome other acute diſeaſes.

We are next preſented with 316 aphoriſms in Latin, ſelected from the writings of Hippocrates, under the title of "*De preſagienda in* "*acutis vita et morte ægrotantium, ſelectæ Hippo-* "*cratis ſententiæ.*"

The author's notes, which fill 69 pages, printed in a ſmaller type than the reſt of the work, are placed at the end of the volume.

 Thoſe

Thoſe at the bottom of a page (and they are in no ſmall number) are by the Engliſh editor, and ſerve greatly to illuſtrate different parts of the work.

In a note to a paſſage on rheumatiſm the author obſerves, that the acute rheumatiſm occurs neither in old age nor in infancy. He has indeed, he tells us, though very rarely, ſeen it in ſubjects of twelve or thirteen years of age, but then it was of leſs violence and duration than it is in patients who are paſt twenty. The chronic rheumatiſm, he has likewiſe obſerved to be more frequent in young people than in thoſe in more advanced life.

The author differs from De Haen and others on the ſubject of critical days, the exiſtence of which he altogether denies. He diſcuſſes this matter with conſiderable ingenuity in a long note.

